# Bone conducted responses in the neonatal rat auditory cortex

**DOI:** 10.1038/s41598-021-96188-9

**Published:** 2021-08-18

**Authors:** Roman Makarov, Mikhail Sintsov, Guzel Valeeva, Pavel Starikov, Dmitriy Negrov, Roustem Khazipov

**Affiliations:** 1 Laboratory of Neurobiology, Kazan Federal University, Kazan, Russia 420008; 2 The School of Electronics, Photonics and Molecular Physics, Moscow Institute of Physics and Technology, Moscow, Russia 117303; 3 INMED, INSERM UMR1249, Aix-Marseille University, 13273 Marseille, France

**Keywords:** Developmental biology, Neuroscience, Physiology

## Abstract

Rats are born deaf and start hearing at the end of the second postnatal week, when the ear canals open and low-intensity sounds start to evoke responses in the auditory cortex. Here, using μECoG electrode arrays and intracortical silicon probe recordings, we found that bone-conducted (BC) sounds evoked biphasic responses in the auditory cortex starting from postnatal day (P) 8. The initial phase of these responses, generated by thalamocortical input, was followed by intracortical propagation within supragranular layers. BC-evoked responses co-localized with the responses evoked by electrical stimulation of the cochlea and the deepest layers of the inferior colliculus prior to onset of low-threshold hearing (P13), as well as with the responses evoked by high-frequency (30 kHz) low-intensity (70 dB) air-conducted sounds after that. Thus, BC signals reach high-frequency processing regions of the auditory cortex well before the onset of low-threshold hearing, reflecting early integrity of the auditory system.

## Introduction

The auditory system—a sensory system for hearing—converts air-conducted (AC) sounds of different frequencies by cochlear hair cells into a pattern of neural impulses which are conveyed in a tonotopic manner through several relay stations towards the auditory cortex^1^. In addition to AC stimuli, cochlear hair cells can also be activated by bone-conducted (BC) signals generated by direct mechanical stimulation of the skull, which can reach the cochlea bypassing the outer and middle ear^2–4^. Development of the auditory neural pathways relies on genetic programs as well as activity-dependent mechanisms which participate in the formation and refinement of developing neuronal circuits^5–9^. Considerable evidence indicates that the activity-dependent development in the auditory system proceeds through two phases which are separated by the onset of hearing^10^. The onset of hearing delineates the opening of the so-called “critical period”, during which exposure to environmental sounds of different frequencies may cause life-long alterations in the tonotopic organization of auditory cortical maps^11–13^. Prior to hearing onset, development of auditory circuits is supported by spontaneous activity generated within the cochlea^14–16^. Remarkably, during this prehearing period, spontaneous cochlear bursts of activity propagate through all auditory relay stations up to the auditory cortex, and demonstrate tonotopic segregation^17,18^. Thus, the onset of hearing is a critical milestone in auditory cortex development.

Despite that, the exact timing of hearing onset has not been clearly defined. Sensitivity of the developing auditory system to sounds depends highly on their intensity, therefore, onset of high-threshold hearing considerably precedes onset of low-threshold hearing^9^. Low-intensity sounds evoke responses in the rat auditory cortex from P11–13^12,13^, at the time when the ear canals open. The propagation of air-conducted sounds to the cochlea at younger ages is limited by the immaturity of the outer and middle ear, therefore, the maturation of the auditory periphery, such as the opening of the auditory meatus, middle ear mesenchyme resorption, and ossification of ossicles, is considered crucial for the onset of low-threshold hearing^19–22^. Yet, cochlear sensitivity to high-intensity stimuli emerges earlier, as evidenced in rats by the appearance of cochlear microphonic (CM) potentials at P8–9^23^, auditory brainstem responses (ABRs) to BC stimuli at P7–8^19^, and behavioural startle responses to high-intensity sounds at P10^24,25^. Similarly in ferrets^26^ and mice^27^ subplate neurons respond to AC sound well before ear opening. Taking into account the ability of spontaneous cochlear bursts to trigger activity in the auditory brain centers already at birth^17,28^, we hypothesised that BC stimuli, which bypass the immature air-conducting pathways, would also be capable of evoking responses in the auditory cortex earlier than the onset of low-threshold hearing.

In the present study, we addressed this hypothesis by exploring the emergence of responses to BC stimuli in the auditory cortex, and by characterizing their spatial–temporal features during the postnatal period using a combination of μECoG epidural recordings and intracortical recordings with silicon probes.

## Results

We used epidural electrocorticographic (μECoG) and intracortical silicon probe recordings to examine the developmental profile and spatial–temporal characteristics of responses evoked by BC stimuli in the auditory cortex of rats aged from P6 to P48 (total n = 25 rats), and compared them with the development of responses evoked by high-intensity acoustic shock waves (ASW), low-intensity AC auditory stimuli as well as with responses evoked by electrical stimulation of the ipsilateral cochlea and the inferior colliculus (IC). The two latter types of stimulation were particularly instructive for ensuring recordings from the auditory cortical areas during the pre-hearing period when AC-evoked responses are not yet present.

### BC stimuli evoke a biphasic response in the auditory cortex of neonatal rats

**Figure 1:**
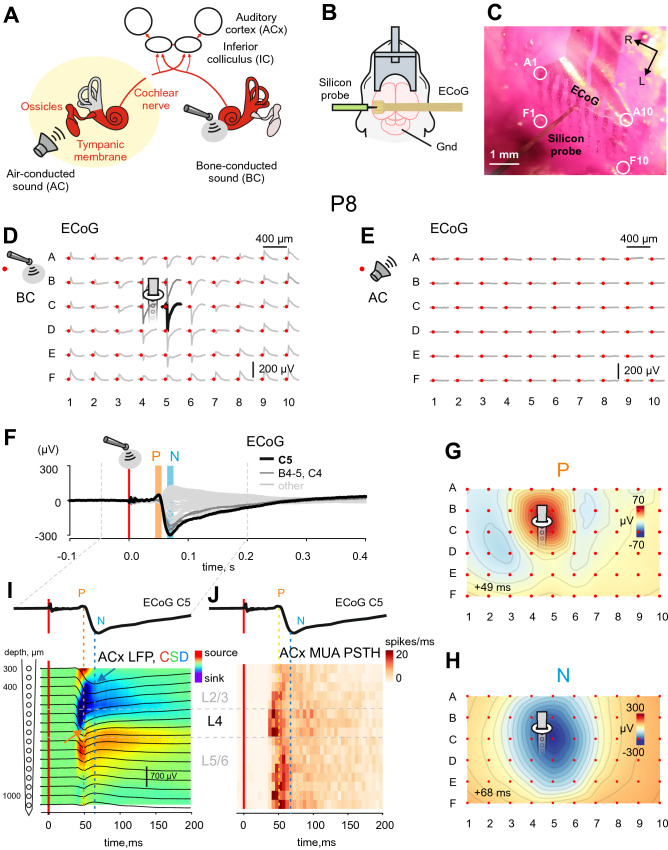
Bone conducted responses in the auditory cortex of neonatal rats. (**A**) Diagram illustrating the flow of activity evoked by air-conducted (AC) sound (left) and bone-conducted (BC) sound (right) through the auditory system (adapted from Babola et al.^17^). (**B**) Experimental setup. Cortical evoked responses recorded in head-restrained urethane-anesthetized rat pups (P6–P16) using a μEGoG electrode and a linear silicon probe. (**C**) Microphotograph of an epidural μECoG electrode array (grid 6 × 10, rows A–F, columns 1–10, vertical and horizontal separation between electrodes 400 μm; positions of corner electrodes are indicated by white circles) placed over the auditory cortex (ACx) in the left hemisphere of a P8 rat and a silicon probe (16 channels, separation distance 50 μm) inserted in the vicinity of the C5 μECoG electrode. R, rostral, and L, lateral. (**D**–**E**) Average responses evoked by BC (**D**) and AC (**E**) stimuli on a μECoG array in the ACx of a P8 rat. The stimulus is indicated by a red dot. Note that AC stimuli fail to evoke any response, whereas BC stimuli evoke robust biphasic responses maximal at the C5 electrode (black trace). (**E**–**J**): average response to 44 BC stimuli. (**F**) Biphasic BC-evoked response on an expanded time scale. Note the early positive (P) and delayed negative (N) components. (**G**–**H**) Spatial map of P (**G**) and N (**H**) component amplitudes at 42 ms and 62 ms after BC stimulus, respectively. Grey lines show 10% amplitude decrements. (**I**–**J**) Concomitant μECoG and silicon probe recordings of local field potential (LFP, black traces) overlaid on a current source density (CSD) map (**I**) and multiunit activity (**J**) at different depths of the ACx. Above, corresponding ECoG traces at the C5 electrode. Note that on (**I**) the early P component of the ECoG BC-evoked response corresponds to the maximum negativity of the response and the early current sink in L4 (orange arrow), whereas the N component matches the LFP signal in L2/3 (blue arrow).

**Figure 2:**
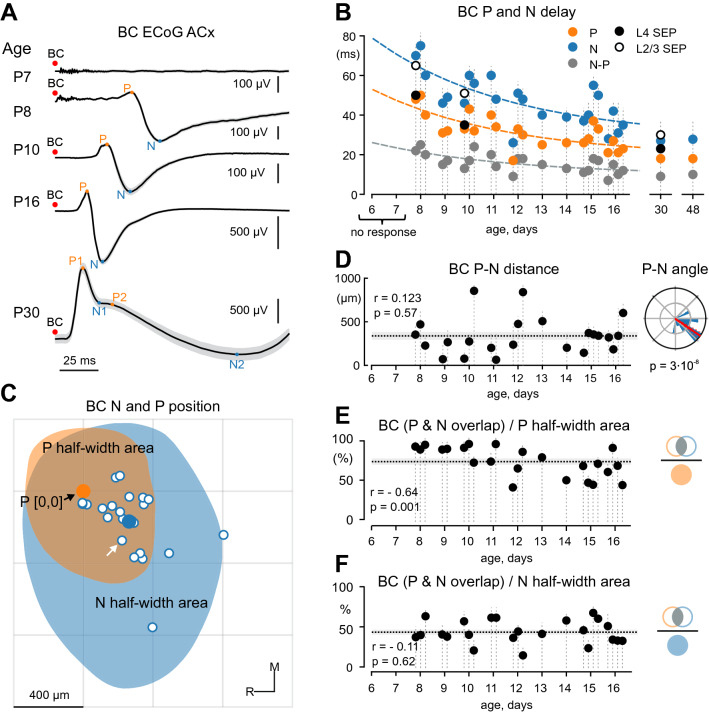
Developmental changes in BC-evoked responses in the auditory cortex. (**A**) Examples of responses evoked by bone-conducted (BC) stimuli in the auditory cortex (ACx) recorded by μECoG array at different postnatal ages. At P7 (top trace), BC stimuli fail to evoke any response in the ACx. Starting from P8 onwards, BC stimuli evoke biphasic responses composed of P and N components (prospective P1 and N1 components in adults), and the response delays decrease with age. (**B**) Developmental changes in delays of the P (orange) and N (blue) components of ECoG responses from BC stimulus, and the delay between the P and N peaks (gray). Dashed lines show exponential fits. Delays for L4 SEP and L2/3 SEP of intracortical responses are indicated by filled and open circles. Vertical dashed lines represent individual animals (n = 25). Note that no response is evoked at P < 8 (n = 2, P6–7 rats). (**C**) Spatial distribution of the N component relative to the P component of the BC-evoked responses to scale of the conceptual electrode grid. N peaks for individual animals (white dots, n = 23) are placed around centred P peaks (orange dot). Mean N position is denoted by the blue dot. Example half-width areas for the P (orange) and N (blue, white arrow indicates correspondent peak) components are shown for an individual P8 animal. R, rostral, and M, medial. (**D**) Distance between P and N peaks as a function of age. The horizontal dashed line shows mean value, shaded area shows SEM. Right, a circular statistical plot of the position of N peaks relative to P peaks. Note that N peaks are located caudo-laterally to P peaks. (**E**–**F**) Overlap of P and N half-width areas relative to P (**E**) and N (**F**) half-width areas. The dotted line represents the mean value, gray shading—SEM.

Responses to BC stimuli were recorded in head-restrained neonatal rats under urethane anesthesia using 60-channel μECoG arrays placed epidurally over the auditory cortex (average centre: 4 mm caudally, 7 mm laterally from Bregma) (Fig. 1A–C). Example response to BC mechanical stimuli applied to the skull through the animal head fixation system (Supplementary Fig. S1) in a P8 rat is shown on Fig. 1D. Biphasic BC-evoked responses were characterized by early positive P and delayed negative N components (Fig. 1F). The P component typically was of smaller amplitude and had a shorter duration than the N component. Modest spread over the cortical surface was observed for the N component (Video 1). The P and N components were largely co-localized (Fig. 1G,H), although the N peak was slightly shifted in the caudolateral direction from the P peak (see also Fig. 2C). Besides that, the P component occupied a smaller cortical area compared to the N component (see also Fig. 2E,F). Concomitant ECoG and intracortical recordings revealed that the P peak corresponds to the maximal sensory-evoked potential (SEP) amplitude and the earliest sink in L4 of the auditory cortex (Fig. 1I) as well as the maximal spiking frequency (Fig. 1J) in the thalamo-recipient L4 and L5/6 border, whereas the N peak corresponds to the maximal SEP amplitude in L2/3 (Fig. 1I). Therefore, we concluded that the P component represents activation of thalamo-cortical synapses, whereas the N component likely reflects transfer of excitation from L4 to L2/3 and further horizontal spread of excitation through the supragranular layers. It should be noted that AC sounds of low intensity (500 ms, 70 dB, 1–40 kHz) failed to evoke any detectable response (Fig. 1E) at this age.

### Developmental changes in BC-evoked responses in the auditory cortex

We further addressed developmental changes in the temporal and spatial organization of BC-evoked responses through the postnatal period. At P6–7, BC stimuli failed to evoke responses in the auditory cortex (Fig. 2A). Starting from P8, BC stimuli reliably evoked biphasic P–N responses in the auditory cortex in all animals, and the delay from stimulus decreased with age (Fig. 2A,B). Latency of both P (*r* = − 0.74, *p* = 0.00002, Supplementary Table 1) and N (*r* = − 0.75, *p* = 0.000015, Supplementary Table 1) peaks from the stimulus, as well as the delay between P and N peaks (*r* = − 0.71, *p* = 0.00008, Supplementary Table 1) decreased with age to attain near-adult values by P15–16 (Fig. 2A,B, n = 25 rats). Also, the developmental trajectory of the P and N peaks from P8 to P16 projected to the P1 and N1 peaks of BC-evoked responses at P30 (Fig. 2A, bottom trace; see also Supplementary Fig. S2). This suggests that the P and N components of BC-evoked responses (as well as those of ASW and AC–evoked responses, see below) in neonatal animals are precursors of the P1 and N1 components in adults. Besides the changes in temporal characteristics BC-evoked responses demonstrated an increase in the amplitude of the P (*r* = 0.48; *p* = 0.02, Supplementary Table 1) and N (*r* = 0.42, *p* = 0.045, Supplementary Table 1) peaks (n = 23 P8–16 rats) with age. It is of note that the late P2 and N2 response components were not present in P8–P16 rats.

Developmental changes in the spatial organization of the P and N components were further explored through two approaches. First, we examined the relative positions of the P and N peaks over the cortical surface. P peaks were centred for all animals and the distribution of N peaks around this central P-point was analyzed (Fig. 2C). The distance between P and N peaks was on average 339 ± 44 μm and did not show any change with age (*r* = 0.123, *p* = 0.58, Supplementary Table 1; Fig. 2D, left). Following the conversion of cartesian peak coordinates to polar coordinates, the Rayleigh test revealed that N peaks were non-uniformly distributed (*p* = 0.3 × 10^−8^) and located in the caudolateral direction relative to P peaks with a mean angle of − 35 ± 6° (where 0° corresponds to the caudal direction; Fig. 2D, right). No age-dependent differences were observed for the P–N angles (*r* = 0.27, *p* = 0.21, Supplementary Table 1). Second, we also analysed the spatial distribution of P and N half-width areas, which are the cortical regions where the response amplitude was higher (lower in case of the N peak) than half of the peak amplitude. We found that the half-width area of the BC-evoked P component was largely nested within the larger N half-width area (Fig. 2C). Intersection of the P and N half-width areas occupied 76 ± 4% of the P half-width area (n = 23 rats). The larger N component spread out of the intersection area in the caudolateral direction with the intersection of the P and N half-width areas occupying 48 ± 3% of the N half-width area (n = 23 rats). We also found that the P half-width area increased in size with age (*r* = 0.45, *p* = 0.03), whereas the N half-width area did not change (1.11 ± 0.07 mm^2^; *r* = 0.2, *p* = 0.31, n = 23 rats, Supplementary Table 1). Besides that, the portion that the intersection of the P and N half-width areas constitutes from the P half-width area decreased with age (*r* = − 0.64, *p* = 0.001, n = 23 rats; Fig. 2E), however there was no such dependence for the N half-width area (*r* = − 0.11, *p* = 0.62, n = 23 rats; Fig. 2F).

### BC-evoked responses co-localize with the ASW and AC-evoked responses

**Figure 3:**
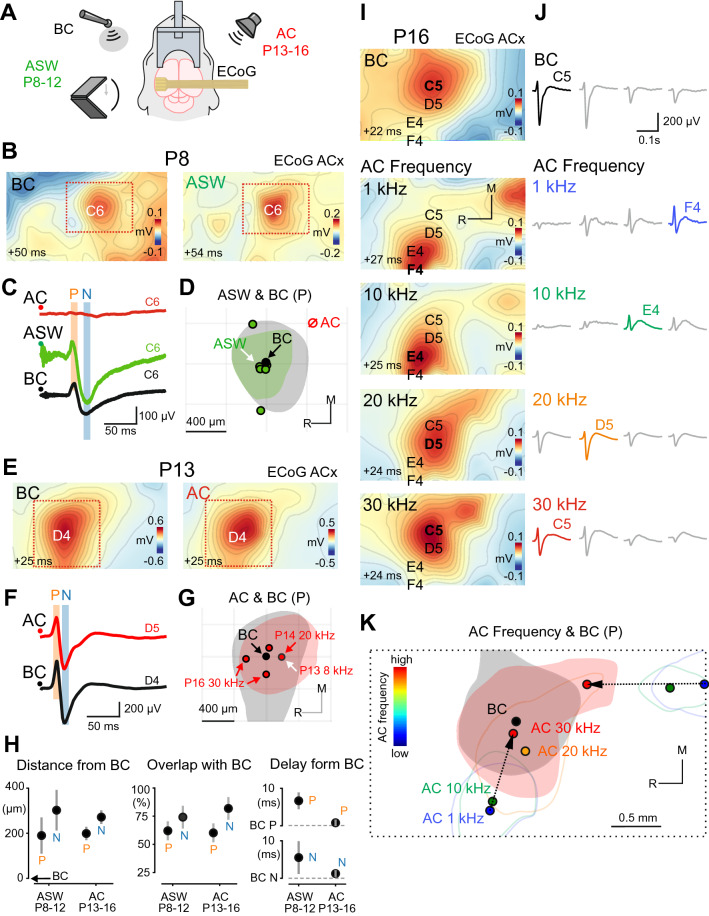
BC-evoked responses co-localize with the AC and ASW-evoked responses. (**A**) Experimental setup: μECoG arrays placed over the auditory cortex are used to record responses evoked by bone-conducted (BC) stimuli, auditory shock waves (ASW) of high intensity (110 dB) produced by a clapper in P8–12 animals (prior to the onset of low-threshold hearing), and by air-conducted (AC) sounds of low intensity (500 ms, 70 dB, 1–30 kHz). (**B**) Spatial maps of the P component of BC and ASW-evoked responses in a P8 animal (the youngest animal in wich ASW stimuli evoked cortical responses). The onset of the 2 ms time window in which the signal was averaged is shown in the lower-left corner. (**C**) Corresponding ECoG traces on the C6 electrode. Note that low-intensity AC stimuli do not evoke any detectable cortical response. (**D**) Positions of ASW-evoked responses (green dots) relative to BC-evoked response (black dot) for P peaks. Pooled data from 6 P8–12 animals. Half-width areas are shown for the animal on (**B**) and (**C**) (correspondent peak is indicated by the white arrow). (**E**–**G**) Responses evoked by BC and AC stimuli in a P13 animal (the youngest animal in wich AC stimuli evoked cortical responses). The layout is similar to (B-D). Pooled data from 4 P13–16 animals. (**H**) Left, latencies of ASW or AC-evoked responses relative to BC-evoked responses. Middle, distances between peaks of ASW or AC-evoked responses, and peaks of BC-evoked responses. Right, the overlap between BC-activated and ASW or AC-activated half-width areas normalized to the size of the BC half-width area. (**I**) Spatial maps of BC (top) and AC-evoked (below) responses at different sound frequencies (1–30 kHz). The onset of the 2 ms time window in which the signal was averaged is shown in the lower-left corner. (**J**) ECoG traces on the channels with maximal amplitude chosen for each map in (**I**). (**K**) Positions of AC responses for different sound frequencies (colored dots) relative to BC-evoked responses (black dot) for P peaks are shown for the animal on (**I**) and (**J**). Note that the half-width area of the BC-evoked response (black fill) overlaps with the half-width area of the AC-evoked response on 30 kHz (red fill), whereas two tonotopic gradients (black arrows) of AC-evoked responses converge towards the position of BC-evoked response.

We further compared the spatial–temporal characteristics of the responses evoked by BC and auditory stimuli (Fig. 3**,** see also Supplementary Fig. S3). We found that AC stimuli of low intensity (70 dB SPL) failed to evoke responses at ages earlier than P13. To reveal whether sound stimuli propagating through the air medium can in principle evoke cortical responses at these ages we attempted to present as intense AC stimuli as possible. We hypothesized that such stimuli could overcome damping in the immature ear structures and be conducted to the hair cells to evoke cortical responses. High intensity auditory stimuli were provided by air-conducted shockwaves, as a shockwave transmits all its energy within a microsecond pulse generating vast pressure. Indeed, AC stimuli of high intensity (~ 110 dB SPL) presented in a form of ASWs evoked cortical responses with electrographic appearance and topography very similar to that of BC-evoked responses starting from P8 (Fig. 3B) (n = 6 P8–12 animals). The mean distance between the P peaks of BC and the ASW-evoked responses was 184 ± 81 μm (N peaks: 297 ± 91 μm; Fig. 3D,H, left) without any preferable direction in the position of BC and ASW P peaks (*p* = 0.12; N peaks: *p* = 0.69). The mean absolute area for the P peak of ASW responses was 0.44 ± 0.06 mm^2^ (N peak: 1.13 ± 0.1 mm^2^). Intersection of the BC and ASW-evoked P half-width areas over the BC P half-width area was 62 ± 8% (N: 74 ± 10%; Fig. 3D,H, middle). The P peak of ASW-evoked responses was slightly delayed from the P peak of BC-evoked responses by 6.7 ± 2.3 ms (N peaks: 5.5 ± 4.4 ms ; Fig. 3C,H, right). Together, these findings point to similarities in the developmental emergence and spatial–temporal organization of BC and ASW–evoked responses starting from P8.

Next, we performed stimulation by AC sounds at low intensity (70 dB SPL) in the frequency range 1–40 kHz (Fig. 3A). In keeping with the results from previous studies^12^, AC sound of low intensity reliably evoked cortical responses starting from P13 (n = 4 P13–16 animals). Another characteristic feature of cortical AC-evoked responses^12,13^ that we observed was tonotopic organization with more prominent tonotopic maps in P16 animals than in P13–14. We found that starting from P14 BC-evoked responses are co-localized with responses to a high frequency AC sound (20–30 kHz). The mean distance between the P peaks of BC and the closest AC-evoked responses was 193 ± 30 μm (N peaks: 266 ± 31 μm; Fig. 3E,H, left) with no preferable relative position of the AC and BC P peaks (*p* = 0.96, N peaks: *p* = 0.89). The mean absolute area for the P peak of AC responses was 0.75 ± 0.14 mm^2^ (N peak: 1.37 ± 0.22 mm^2^). Intersection of the BC and AC-evoked P half-width areas over the BC P half-width area had a mean value of 60 ± 5%, (N: 82 ± 7%; Fig. 3G,H, middle). The P peak of AC-evoked responses was delayed from the P peak of BC-evoked responses by 0.667 ± 0.72 ms (N peaks: 1.0 ± 0.68 ms; Fig. 3F,H, right). Together, the similar spatial and temporal characteristics of BC and AC-evoked responses suggest that BC stimuli evoke responses in the auditory cortical areas.

No responses were observed to the stimulation at 40 kHz, but stimulation at other frequencies (30, 20, 10, 1 kHz) reliably evoked cortical responses in P13–P16 animals. In all examined animals (n = 4 rats) BC-evoked responses were co-localized with AC-evoked responses. However, in P16 animals (n = 2) BC-evoked responses were closest to the responses to the highest AC sound frequency (30 kHz) and furthest from the responses to the lowest AC sound frequency (1 kHz) (Fig. 3I–K). The half-width areas of BC-evoked responses also largely overlapped with the half-width areas of the responses to 30 kHz AC sound but did not overlap with the half-width areas of the responses to the 1-kHz AC sound (Fig. 3K). Cortical responses to AC sound demonstrated frequency-dependent localization as illustrated by the example responses from a P16 animal on Fig. 3. These responses were organized in two opposite frequency gradients (low-to-high) with one running in the caudo-rostral direction and the other in the latero-medial direction (Fig. 3K, black arrows). Both gradients were considerably reproduced among animals (caudo-rostral in n = 3 rats; latero-medial in n = 2 rats).

### BC-evoked responses co-localize with the cochlea- and IC-evoked responses

**Figure 4:**
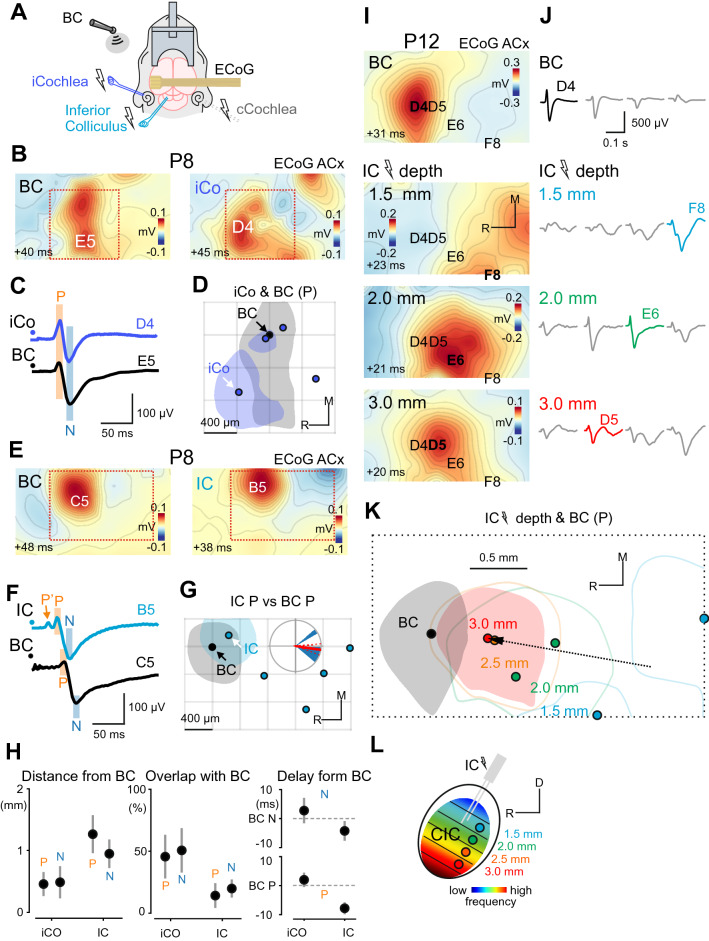
BC-evoked responses co-localize with responses to ipsilateral cochlea (iCO) and inferior colliculus (IC) stimulation. (**A**) Experimental setup: μECoG arrays placed over the auditory cortex are used to record responses evoked by bone-conducted (BC) stimuli, and by electrical stimulation (50 µs, 60 V, 0.1 Hz) of the iCo and the IC. (**B**) Spatial maps of the P component of BC and iCo- evoked responses in a P8 animal (different to Fig. 3B–D animal). The onset of the 2 ms time window in which the signal was averaged is shown in the lower-left corner. (**C**) Corresponding ECoG traces on the channels with maximal amplitude for each map in (**B**). (**D**) Positions of iCo-evoked responses (blue dots) relative to BC responses (black dot) for P peaks. Half-width areas are shown for the animal on (**B**) and (**C**) (correspondent peak is indicated by the white arrow). Pooled data from 4 P8–16 animals. (**E**–**G**) Responses evoked by BC and IC stimuli in a P8 animal. The layout is similar to (**B**–**D**). Pooled data from 5 P8-15 animals. (**H**) Left, latencies of iCo and IC-evoked responses (P and N peaks) relative to BC-evoked responses. Middle, the distances between the peaks of iCo and IC-evoked responses and the peaks of BC-evoked responses. Right, the overlap between BC-activated and iCo or IC-activated half-width areas normalized to the size of the BC half-width area. (**I**) Spatial maps of BC (top) and IC-evoked responses at different depths of stimulation (1.5–3 mm). (**J**) ECoG traces on the channels with maximal amplitude for each map in (**I**). (**K**) Positions of IC-evoked responses at different depths of stimulation (indicated by color dots) relative to BC-evoked responses (black dot) for P peaks are shown for the animal on (**I**) and (**J**). Note that the half-width area of the BC-evoked response (black fill) overlaps with the half-width area of the IC-evoked response at 3.0 mm (red fill), whereas the tonotopic gradient (black arrow) of IC-evoked responses is directed towards the position of the BC-evoked response. (**L**) Diagram illustrating the distribution of tonotopic layers across the depth of the IC. *CIC* Central nucleus of the inferior colliculus, *R* Rostral, *D* Dorsal.

We further performed electrical stimulation of the ipsilateral cochlea (n = 4 rats) and the ipsilateral IC (n = 5 rats) using bipolar electrodes (50–100 μs, 60–70 V, 0.1 Hz; Fig. 4, see also Supplementary Fig. S4). The mean distance between the P peaks of BC and the cochlea-evoked responses was 459 ± 194 μm (N peaks: 488 ± 260 μm; Fig. 4B,H, left) with no preferable relative position of the cochlea- and BC-evoked P peaks (*p* = 0.456; N: *p* = 0.3). The mean absolute area of the P peak of cochlea-evoked responses was 0.58 ± 0.06 mm^2^ (N peak: 0.85 ± 0.15 mm^2^). The intersection of the BC and cochlea-evoked P half-width areas over the BC P half-width area was 46 ± 18% (N: 51 ± 18%; Fig. 4D,H, middle). The P peak of cochlea-evoked responses was delayed from the P peak of BC-evoked responses by 2.0 ± 2.48 ms (N peaks: 2.75 ± 4.37 ms; Fig. 4C,H, right). The similar spatial and temporal characteristics of BC and cochlea-evoked responses suggest that BC stimuli also evoke responses in the auditory cortical areas during the pre-hearing period.

Stimulation of the IC reliably evoked cortical responses starting from P6 (the earliest examined age). In a P8 animal the BC and IC-evoked responses were co-localized (Fig. 4E–G). However, in P10–13 animals the localization of the IC-evoked responses depended on the depth of stimulation (Fig. 4I–L). The mean distance between the P peaks of BC and the closest IC-evoked responses was 1263 ± 306 μm (N peaks: 947 ± 231 μm; Fig. 4G,H, left) with IC-evoked responses located in the caudal direction relative to the P peaks with a mean angle of -8 ± 12° (*p* = 0.007; N peaks: -8 ± 19°, *p* = 0.03; Fig. 4G). The mean absolute area for the P peak of IC-evoked responses was 1.01 ± 0.28 mm^2^ (N peak: 1.16 ± 0.25 mm^2^). The intersection of the BC and IC-evoked P half-width areas over the BC P half-width area was 14 ± 10% (N: 20 ± 7%; Fig. 4G,H, middle). The P peak of IC-evoked responses was earlier than the P peak of BC-evoked responses by 7.8 ± 2.06 ms (N peaks: 4.25 ± 2.94 ms; Fig. 4F,H, right). Another interesting observation was that the stimulation of the IC reliably evoked short latency antidromic spikes in the presumptive L5b of the auditory cortex (Supplementary Fig. S5) which resulted in the emergence of the P’ ECoG component preceding the P component (Fig. 4F). Since these antidromic spikes in adult animals signify the existence of cortico-collicular projections^29^, our findings suggest that this type of connections is present prior to the onset of low-threshold hearing.

In addition to the tonotopic maps obtained for responses to AC-sound, stimulation of the IC also evoked cortical responses in a topographic manner depending on the depth of stimulation (n = 3 rats) (Fig. 4K). This activation pattern is similar to the one in adult rats, in which each layer of the central nucleus of IC (CIC) projects through the thalamus to a particular part of the auditory cortex. Moreover, this laminar pattern gives rise to a frequency gradient, in a way that higher frequencies are represented in deeper parts of the CIC^1^ (Fig. 4L). Therefore, we conclude that our findings reflect early tonotopic organization of the collicular-cortical pathway. Interestingly, the BC-evoked responses were localized closer to the responses to stimulation of the deepest (high-frequency) part of the IC and further from the responses to stimulation of the superficial part (Fig. 4K). This observation suggests that the BC-evoked responses are localized in high-frequency processing auditory cortex regions.

### Bilateral cochlear ablation eliminates BC-evoked responses

**Figure 5:**
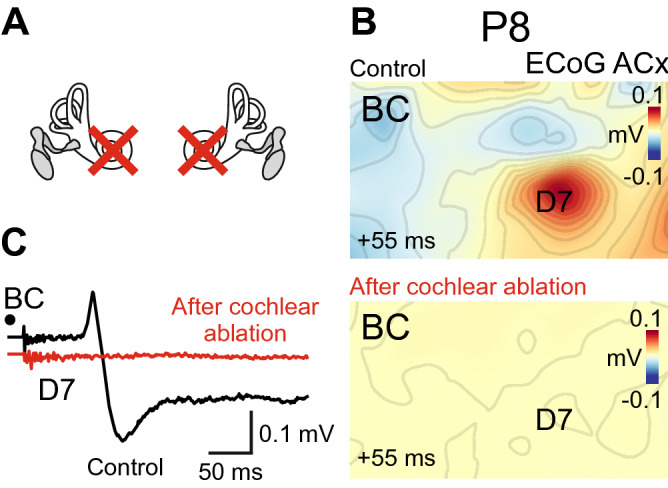
BC-evoked responses can be eliminated by bilateral cochlear ablation. (**A**) Diagram illustrating bilateral cochlear ablation. (**B**) Spatial maps of BC-evoked responses before (top) and after (bottom) bilateral cochlear ablation for a P8 animal. The onset of the 2 ms time window in which the signal was averaged is shown in the lower-left corner. (**C**) Corresponding ECoG traces on the D7 electrode. Note that BC stimuli do not evoke any detectable cortical response after the ablation.

We further investigated whether the BC-evoked responses involve cochlear activation (Fig. 5) in two animals (P8 and P16). We found that after bilateral cochlear ablation (Fig. 5A; see also Supplementary Fig. S6) BC stimuli failed to evoke any detectable cortical responses in the cortical areas where the responses to BC-sound were observed in control recordings (Fig. 5B,C). Amplitude of the P peak dropped to 0.033% and 0.02% of the control values in a P8 and a P16 animal (N peak: 0.06% and 0.014%) respectively. These results suggest that BC-evoked responses involve cochlear activation and propagate through the auditory system up to the neocortex.

## Discussion

In the present work we addressed the development of sensory-evoked activity in the rat auditory cortex. Our main finding is that BC stimuli evoke responses in the rat auditory cortex starting from P8 and that similar responses can also be evoked by excessively high-intensity (> 100 dB SPL) ASW stimuli. These responses involved initial activation of thalamorecipient areas followed by caudal-lateral intracortical spread of activity, were largely co-localized with the responses evoked by electrical stimulation of the ipsilateral cochlea and IC, and required cochlear activation. Besides that, BC-evoked responses tended to activate high-frequency sound processing areas in the developing auditory cortex. Altogether, these findings suggest that the onset of sensory-evoked activity in the auditory cortex occurs not as a sharp developmental switch, but rather as a transition from high-threshold to low-threshold hearing over the course of the second postnatal week and that the developmental changes in cortical responses during this period involve maturation of both the sensory periphery and central auditory-processing networks.

### Early biphasic responses in the auditory cortex

Early responses, recorded from the cortical surface using μECoG electrodes, were observed as biphasic evoked potentials with the initial positive P and late negative N components. Using coupled μECoG and intracortical recordings we revealed the relationship between superficial and intracortical signals. We found that the superficial P component represents the passive source from the earliest sinks in the thalamorecipient layers L4 and L3b. It is also possible that thalamic input to the L5/6 neurons, which have long apical dendrites, contribute to the passive source generation in superficial layers. The influence of deep activity on the superficial signals was also supported by the fact that antidromic spikes evoked by electrical stimulation of the IC give rise to the P' component of the ECoG signal. As current sinks mainly reflect the activation of synaptic connections, they allow localizing the populations of neurons receiving strong input in a given moment of time. We thus conclude that the BC-evoked signals enter the cortex through the thalamorecipient layers. We also found that the late superficial N peak corresponded to the N peaks in L2/3, which is in accordance with the previous reports that waveforms of EEG signals considerably resemble LFP signals from the superficial cortical layers^30^. It is of note, however, that peaks do not map onto distinct generators in a simple one-to-one manner, but rather each peak is usually determined by more than one separate generator and, moreover, even the rising and falling stage of a peak might originate from different sources^30–32^.

The spatial location of the P and N peaks was slightly different, with the N peak located caudo-laterally relatively to the P peak. Moreover, we observed propagation of the N component in the caudo-lateral direction. As the superficial N peak is thought to originate from activity in the supragranular layers it may represent intracortical propagation of activity by connections within L2/3. Indeed, our observation is in accordance with previously reported activity propagation within isofrequency stripes in the adult primary auditory cortex. It was shown that in adult guinea pigs, activity first enters the dorsal part of a cortical isofrequency stripe through thalamocortical connections and then propagates along it to the ventral part through L2/3, without significant involvement of the thalamus^33^. However, we did not observe widespread propagating waves in the auditory cortical areas present in adult rats^34^. We, thus, conclude that the intracortical propagation of activity through the superficial layers within the primary areas develops prior to the onset of low-threshold hearing. Horizontal connections within superficial layers play an important role in representing complex auditory objects^35^ and, therefore, constitute important targets of functional tuning during the critical period of enhanced plasticity. This might explain their early emergence. Together, the depth profile of activity and the spatial spreading of the N component suggest that BC-evoked signals enter the auditory cortex via thalamocortical connections and spread vertically through L4–L2/3 connections within the thalamorecipient regions expressing both P and N components and horizontally via supragranular layers to adjacent P-lacking N regions.

The P and N components observed in neonatal and juvenile ($\le$ P16) animals displayed a developmental decrease in time delays from the stimulus onset, and their developmental trajectory approximated the P1 and N1 peaks by one month of age. These early sensory-evoked responses in $\le$ P16 auditory cortex lacked the delayed P2, N2 and P3 components characteristic of auditory processing in the adult cortex, however^36–38^. Current source density and multiunit activity analysis of intracortical responses in a P30 animal indicated that the P2 peak corresponds to the sink in L5, the N2 peak represents a K-complex, and the P3 peak is a passive source from a deep sink of the sensory evoked cortical UP-state. The lack of these late components in ≤ P16 animals likely reflects immaturity of the intracortical circuitry involved in the generation of these activity patterns^10^.

### Developmental periods of hearing in the auditory cortex

**Figure 6:**
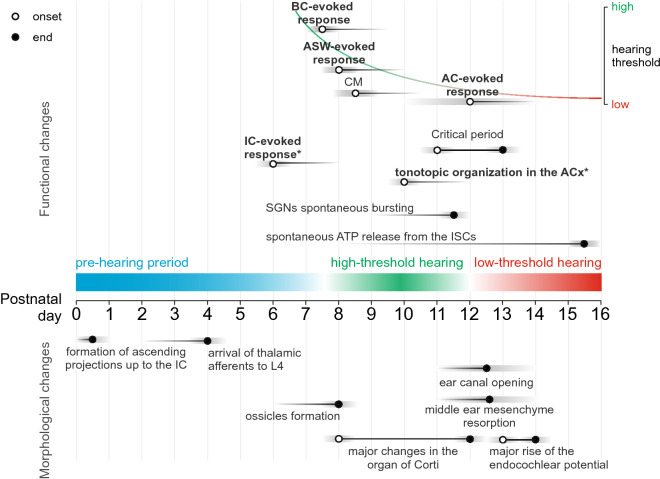
Three epochs of functional maturation in the rat auditory system. Developmental timeline from P0 (day of birth) to P16 is shown in the middle. The major physiological (above) and morphological (below) changes observed in the rat auditory system during this period are indicated by black horizontal lines and circles (onset—open circle, end—filled circle). Three epochs of functional maturation are represented by colored bars. Morphological changes were obtained in: connectivity^10,39^, inner ear^40,41^, and middle and outer ear^19,22,42,43^. Physiological changes were obtained in: spontaneous activity changes^15^, and evoked activity changes^19,23^. Grey bars represent variability of values in reports. The functional changes investigated in this study are written in bold. The asterisk indicates the earliest examined age. *BC* Bone-conducted, *ASW* Auditory shock wave, *CM* Cochlear microphonic, *AC* Air-conducted, *IC* Inferior colliculus, *ACx* Auditory cortex, *SGNs* Spiral ganglion neurons, *ISCs* Inner supporting cells.

Based on the present results and previous reports, we can divide the functional maturation of sensory responses in the rat auditory cortex into three developmental periods: prehearing period (< P8), high-threshold hearing (P8–13) and low-threshold hearing (> P13) (Fig. 6).

#### Prehearing period

In accordance with the previously reported onset of the ABRs to BC stimuli at P7–8^19^ and CM potentials to high-intensity AC stimuli at P8–9^23,42^ the first cortical responses to the BC and ASW stimuli in our study were obtained at P8. Thus, the prehearing period of complete deafness of immaturity lasts until ~ P8, although all elements of neural circuitry conducting signals from the periphery to the cortex including thalamo-cortical connections (to the subplate from E17 and to L4 from P3–4)^10^ are put in place well before this^39^. This is also supported by our observation that electrical IC stimulation reliably evokes responses in the auditory cortex at P6–7.

#### High-threshold hearing

During the period of high-threshold hearing, responses to low-intensity AC sounds are not observed, likely due to the immaturity of the conductive structures of the outer and middle ear. However, sensory stimuli that reach the cochlea (BC, ASW) reliably evoke cortical responses. The decrease of the latency and the increase of the amplitude of these responses described here correlate with the threshold improvement for ABRs evoked by BC stimuli during the second postnatal week^19^ and likely reflect morphological changes in the cochlea occurring between P8 and P12^40^. Along with the maturation of ear structures, neuronal development in the central auditory system can play an important role in the age-related differences reported in this study. The high-threshold hearing period falls within the first phase of the late period of the auditory cortex development^10^. At P8 the auditory cortex in rats already has a clearly defined laminar structure, as the major neuronal migration is completed^44^. The total number of cortical neurons peaks at P7 and then decreases during the second week as cells undergo programmed death. However, the overall size of the cortex continues to increase due to ongoing gliogenesis and maturation of individual neurons, which develop complex morphology through neurite outgrowth^45^. The increase in amplitude and size of the areas expressing the P component can be related to changes in the size of relevant neuronal populations and in the effective size of the primary auditory cortex^46,47^. Along with astrogenesis and neurite outgrowth, synapse formation peaks during the second postnatal week^45^. Nascent synapses undergo changes in their structure and receptors composition, which lead to a decrease in latency and duration of PSPs^10^. The decrease in latency of evoked responses demonstrated in the present work, thus, can be related to developmental changes in synaptic currents, but also to changes in spike initiating and propagating characteristics^48^. Myelination less likely contributes to these changes as it is counterbalanced by an increase in projection length^10^. It was shown that the coarse tonotopic arrangement of the synaptic connections can develop prior to auditory experience, although this process may require spontaneous activity^10^. It is of importance to note that spontaneous auditory cortex activity during the prehearing and high-threshold hearing periods is driven by spontaneous cochlear-driven activity which emerges shortly after birth and stops at the onset of low-threshold hearing along with cessation of spontaneous bursting of the spiral ganglion neurons and ATP release from inner supporting cells^15^. Another possible contributor to age-related differences in bone conduction is the maturation of the craniofacial bones and the system of the middle ear ossicles. Although ossification of the skull in rats and mice ends within two days after birth^49^, the size and composition of its bones continue changing. In mice, the head length has the fastest growth during the first week with additional fast growth between P7 and P21 and slows down more than fourfold after that. The height of the cranial vault also significantly increases between P7 and P14. However, the length and thickness of the frontal, parietal and interparietal bones remain relatively constant at the early postnatal stage, increasing only after P30^50^. The hardness and rigidity of bone tissue depend on the incorporation of minerals into the protein matrix, and the major role in this process plays calcium, the concentration of which in rats increases between P7 and P28^51^, potentially making a bone a better sound conductor. Three centers of ossification are observed in rat middle ear already at birth, however, the ossicles become clearly defined only at P8^52^. The oval window achieves adult-like size by P13, while the tympanic membrane—by P17^53^. Nevertheless, during the high-threshold hearing period, mesenchymal fluid fills the middle ear and prevents the transmission of sounds through the ossicle chain.

#### Low-threshold hearing

While high-intensity auditory signals (ASW) were able to evoke cortical response starting from P8, low-intensity AC stimuli (70 dB SPL) evoked cortical responses starting from P13 in keeping with the developmental auditory threshold improvement^54^ and the fuzzy boundary in the onset of the low-threshold hearing^12,13,19,21,42,43,55^. The onset of low-threshold hearing is conditioned by ear canal opening at P12–13^21,22^, the middle ear mesenchyme resorption relieving the ossicles at P11–14^22,42,43^, and functional maturation of the inner ear with an emergence of the endocochlear potential between P11 and P17 with the steepest increase at P13–P14^41^. The adult-like thresholds and latencies are achieved at around P20 and correlate with the adult appearance of the organ of Corti^19,40,55,56^. The onset of low-threshold AC hearing is a crucial milestone in the auditory cortex development since it manifests the opening of the critical period of activity-dependent plasticity in the rat auditory cortex lasting for about 3 days and resulting in the refinement of sound representation maps^13^.

A similar sequence of developmental events can be observed in other altricial mammalian species, such as ferrets^26^, gerbils^20^ and mice, although their timing can be significantly shifted relative to birth. In mice, the auditory cortex can respond to sounds at P9—earlier than in rats and well before ear canal opening at P11–12^27^. The major morphological changes in the mice cochlea also occur several days earlier than in rats—between P5 and P10^40^. Parallels with human development are not so straightforward, since human fetuses respond to airborne sounds already at mid-gestation that roughly corresponds to the birth in rodents. In preterm humans, ABRs can be recorded starting from the 26 to 28 weeks of gestation^57^ and cortical responses are evoked from 28 weeks^58,59^. Nevertheless, prenatal humans can also have a high-threshold hearing period, when external sounds are attenuated by the womb^9^. Thus, the present study, contributes to the current paradigm shift in the hearing development research, since the onset of hearing being treated now as a gradual process with the periods of high- and low-threshold hearing^9,60^. This is an important clarification, since the early evoked activity has been shown to remarkably influence the functional maturation of the auditory cortex^12,13^. In view of this, earlier ages should be examined on possible experience-dependent alterations in the auditory function.

Yet the exact contribution of early BC and ASW-evoked responses to the development of the rat auditory system remains largely unknown. These stimuli seem to rarely occur in normal raring conditions, however, since in humans BC sound was shown to participate in the perception of the subject's own vocalizations^61,62^, we speculate that pups could be self-stimulated by early vocalizations^63,64^, eating, or other endogenous stimuli days before the onset of sensitivity to environmental sounds. Thus, it is possible that early responses and spontaneous activity co-exist and cooperate during the high-threshold hearing period.

### Tonotopic organization of the BC-evoked cortical responses

The auditory cortex can be divided into the tonotopically organized primary auditory area and anterior auditory field, the tonotopic gradients of which converge in the high-frequency regions, and surrounding non tonotopic secondary regions^65,66^. According to this organization, stimulation by the low-frequency sounds evoked two foci of activity in the auditory cortex, on the opposite ends of the frequency gradients. As the sound frequency increased, the adjacent populations of neurons became activated, until the two foci converged in the high-frequency regions. Several lines of evidence indicate that tonotopic organization of the auditory cortex emerges before the low-threshold hearing onset and that BC-evoked responses primarily activate high-frequency processing areas. Similar tonotopic gradients in AC-evoked responses were observed in juvenile (P16) animals, and the size of the primary auditory area and anterior auditory field inferred from the distances between the responses to the lowest and highest frequency sounds was ~ 1–1.5 mm in length, which is comparable to the size of these areas in adult rats^34,67–69^. BC-evoked responses at P16 were most prominent in the high-frequency (30 kHz) auditory cortex regions. Preferential activation of the high frequency regions during BC-evoked responses likely accounts for the location of the high-frequency processing cochlear basal turn directly on the temporal bone thus receiving more skull-conducted sound energy during BC-stimulation^70,71^. In addition, basal to apical maturation gradient can be observed in some cochlear structures, which possibly makes the basal turn more sensitive during the high-threshold hearing period^9,28^.

We also found that tonotopic propagation of signals from the IC to the auditory cortex is present prior to the onset of low-threshold hearing (P13). In P10–P13 animals, cortical responses to electrical stimulation of the IC shifted more than a millimeter in the caudo-rostral direction with an increase in the stimulation depth in the IC, as the electrode approached the layers where high-frequency sounds are processed. Remarkably, the BC-evoked cortical responses were closest to the responses evoked by stimulation of the high frequency processing deep IC layers. These findings are in keeping with mesoscale calcium imaging studies in P7–8 mice, in which the IC and auditory cortex regions encoding similar sound frequencies exhibited synchronous bursts of spontaneous activity^17^.

## Conclusions

In conclusion, we show that BC stimuli evoke responses in the rat auditory cortex starting from P8, well before the low-threshold hearing onset (at P13) and that similar responses can also be evoked by high-intensity ASW stimuli. Thus, the onset of sensory-evoked activity in the auditory cortex occurs not as a sharp developmental switch, but rather as a protracted process spanning the second postnatal week, that is in general agreement with the fuzzy boundary in the onset of the low-threshold AC hearing^12,13,19,21,42,43,55^. Our results also point to the remarkable level of maturity of the developing auditory system prior to the onset of low-threshold hearing including tonotopic organization, that is in keeping with the tonotopic organization of spontaneous cochlear-driven activity^17,28^, and the presence of intracolumnar and horizontal processing networks.

## Methods

### Ethical approval

The animal experiments were carried out in compliance with the appropriate Animal Research: Reporting In Vivo Experiments (ARRIVE) guidelines. Animal care and procedures were in accordance with EU Directive 2010/63/EU for animal experiments, and all animal-use protocols were approved by the French National Institute of Health and Medical Research (APAFIS #16992-2020070612319346 v2) and the Local Ethical Committee of Kazan Federal University (No. 24/22.09.2020).

### Animal preparation

Wistar rats of both sexes from postnatal day P6 (P0 day of birth) to P48 were used. Inhalation anesthesia was induced with vaporized isoflurane (4%) for about 5 min followed by urethane intraparietal injection (1 g/kg). In addition, lower levels of isoflurane anesthesia (0.5%–1%) were maintained during the surgery depending on the animal’s state. The scalp was removed to expose the skull and the fascia were removed from the surface of the skull. The temporal muscles on the left side of the skull were removed and the neck muscles were detached from the occipital bone. Craniotomy was performed to expose a 4 × 7 mm cranial window above the temporal and parietal bones on the left side leaving the *dura mater* intact. A brass half-ring was attached to the frontal bones using dental cement (Grip Cement, Caulk Dentsply, DE, USA). After surgery the animal was placed on a warmed platform (37 °C) for 20–30 min. The metal half-ring was then attached to a fixation system via a ball joint.

### Custom µECoG electrode arrays

Custom µECOG electrode arrays were designed as 6 × 10 chromium-gold electrode grids on a polyimide film base with 0.4 mm separation between electrodes (Moscow Institute of Physics and Technology, Russia). For manufacturing, polyimide varnish AD-1903 with a polyimide content of 13% was applied on a silicon wafer with a diameter of 4 inches by centrifugation (4000 rpm for 1 min). Then the solvent was evaporated for 10 min at a temperature of 170 °C, followed by thermal imidization. During the imidization, the film was heated in vacuum to a temperature of 350 °C at a rate of 1 °C/min. The thickness of the polyimide coating was 6 μm. Then, the topology of the electrode was formed on the surface of the polyimide film by optical two-layer lithography. The polymer LOR-5B was applied to the polyimide by centrifugation (4000 rpm for 1 min) and dried in the atmosphere at a temperature of 170 °C for 10 min. Then photoresist AZ-1505 was applied (4000 rpm for 1 min) and drying was carried out at a temperature of 100 °C for 2 min. Lithography was carried out on an AML-100 optical lithograph at radiation power 90 mJ. Development was carried out in 0.8% KOH solution at 21 °C for 50 s. The deposition of metal layers of chromium and gold was carried out in one pumping cycle. Chromium was deposited by DC magnetron sputtering at a power of 400 W from a target with a diameter of 75 mm; the deposition rate was 33 nm / min. Since chromium acts as an adhesive sublayer, its thickness was 10 nm. Gold 100 nm thick was deposited by electron beam evaporation in a vacuum. After removal of the resist, metallization remains on the surface of the polyimide in the form of the required topology with connecting tracks and pads without defects at the edges. Then the resulting structure was covered with a second polyimide layer using the same technology. The windows on the contact pads were opened by dry etching through a hard mask.

### Electrophysiological recordings

#### ECoG recordings

Recordings were made using custom thin-film μECOG electrode arrays. Recordings were performed from the left cerebral hemisphere, which in rats has stronger connections with the right (contralateral) ear^1^. The thin-film electrode was applied to the wetted *dura mater* above the auditory cortex. Preliminary recordings were made and, if needed, the film was shifted in such a way that the center of the film was aligned with the center of the area responsive to the BC stimuli. The final position of the center of the μECoG electrode array in P6–P16 animals on average was 4 mm caudally from Bregma and 7 mm laterally from the sagittal suture.

#### Intracortical recordings

Intracortical recordings were performed simultaneously with the surface recordings using linear silicon probes (16 channels, 50 μm separation distance between recording sites, Neuronexus Technologies, USA). The linear probe was placed between the channels of the film electrode with the maximal magnitude of evoked responses to the stimuli to be examined. Specifically, after the positioning of the film electrode, the linear probe was inserted to a depth of 700–1000 μm depending on the animal age.

### Stimulation

#### Bone-conducted sound stimulation

Light taps by a small steel rod (20 cm long, ~ 220 g) were used as the BC mechanical stimuli with the interstimulus interval of 3–5 s. The stimuli were applied manually to the steel table and propagated through the animal head fixation system, consisting of a magnetic stand (Narishige GJ-1, Japan) connected to the brass head mount half-ring. The taps were recorded by a piezo detector and their onsets were further extracted using detection by thresholding. BC stimuli had a broad-band frequency spectrum with the most prominent peaks between 500 and 2000 Hz (Supplementary Fig. 1D) as measured by a microphone (20–20,000 Hz, Shure MV5). In addition to the BC component, these stimuli also produced auditory component, which intensity was of < 70 dB SPL and thus did not exceed the low-intensity AC stimuli (70 dB SPL) as measured by a sound level meter (EXTECH 407,764, USA) in the peak intensity mode. We did not distinguish between the ears, since it was not possible to separate the propagation of the BC stimuli within the skull and present them to a particular ear.

#### Air-conducted sound stimulation

AC sound was presented as 500 ms stimuli in the frequency range of 1–40 kHz (1, 10, 20, 30, 40 kHz) produced by a signal generator (arbitrary waveform generator G6-29, Russia) with the interstimulus interval of 2 s. The speaker was placed within 5 cm from the right (contralateral) ear and the sound intensity of 70 dB SPL was measured by a sound level meter (EXTECH 407,764, USA). Ambient sound intensity was maintained below 40 dB SPL. AC stimuli were presented to the right ear, however, the left ear was not surgically occluded and thus could also receive stimulus yet at lower intensity. High-intensity ASW stimuli (max 110 dB SPL, non-harmonic) were produced with the interstimulus interval of 3–5 s. ASW stimuli were produced by a clapper made of two flat wooden plates of 300 g ~ 10 × 10 cm each attached to the endpoints of one-meter-long metal levers connected via a hinge joint. The clapper had been put into action manually by the experimenter, at a distance of 1.5 m from the animal. The peak intensity of a clap at this distance exceeded 100 dB SPL, as measured by a sound level meter (EXTECH 407,764, USA).

#### Electrical stimulation of the IC

Electrical stimulation of the IC was performed using a bipolar electrode (area = 750 μm^2^, tips separation = 150 μm, tips axial shift = 250 μm; Microelectrodes Ltd., UK). Stimulation of the IC was performed from the ipsilateral (left) side, since neurons in the CIC project largely to the medial geniculate body on the ipsilateral side^1^. For this purpose, a 1–2 mm hole in the intraparietal bone was drilled using a dental drill at 2.0–2.5 mm lateral to the midline and 2.5–3.0 mm caudal to the lambda depending on age and the electrode was inserted (45° to the sagittal plane, 45° to the horizontal plane). Electrical stimuli of 50–100 μs duration and 60–70 V amplitude were applied every 10 s. The depth of the stimulation from the skull surface was changed with 0.5 mm increment from 0.5 to 1 mm (superficial layers) to 2–3 mm (deep layers) depending on age.

#### Electrical cochlear stimulation and cochlear ablation

The surgical approach to the cochlea was based on the posterior tympanum approach via a retroauricular incision^72^. Briefly, a retroauricular incision was made and the external auditory canal was dissected, the tympanic membrane and the ossicles were removed. When the tympanic cavity was exposed, the cochlear wall was perforated using a dental drill (Supplementary Fig. S6) and a bipolar electrode was then inserted into the cochlea closer to the drilled apical end. While the cochlear wall was perforated, we tried to avoid damage to the modiolus and the cochlear nerve fibers. Stimulation of the cochlea was performed using the same bipolar electrodes and stimulation protocol as for the IC. Stimulation of the contralateral and ipsilateral cochlea evoked similar cortical responses, therefore stimulation of the ipsilateral cochlea was further used as an easier procedure. For the bilateral cochlear ablation, both cochleae were destroyed using the dental drill and the remaining cochlear contents were aspirated using a pump (n = 2 rats). All manipulations with the cochlea were conducted after recordings of sensory-evoked responses.

### Data analysis

#### ECoG recordings

Recordings were preprocessed using custom routines written in Python. The raw ECoG signal was downsampled to 2 kHz and split into 1.5 s epochs (0.5 s before stimulus, 1 s after stimulus). Average reference and baseline correction were then applied. Noisy channels were replaced with the averaged values from the neighboring channels. To obtain topographic maps of the signal components (P or N), first, the channel with the maximal amplitude of the component’s peak was defined. Then, the signal amplitude was averaged for each channel within a ± 1 ms window around the peak. Intermediate amplitude values between electrodes were inferred using spline interpolation with a 1 μm step. To further analyze the relative spatial characteristics of responses we defined peak locations as the spatial coordinates of the maximal response amplitude, and the half-width areas where the response exceeded half the peak amplitude. We then converted the Cartesian coordinates of the peaks (x, y) to the polar ones to obtain the P–N distance $r = \sqrt {x^{2} + y^{2} }$ and angle $\theta = tan^{ - 1} \left( {y/x} \right)$.

#### Intracortical recordings

For the analysis of the intracortical recordings the raw signal was also split to 1.5 s epochs. Spikes were detected from the 0.25–4 kHz band-passed signal using Klusta detector and the average PSTHs with a 5 ms bin were obtained. The raw signals were then low-pass filtered and downsampled to 2 kHz to extract the LFP component and current source density (CSD) analysis was applied to eliminate volume conduction and localize synaptic currents. CSD was computed for the averaged signal according to the standard method^73^.

#### Statistics

Statistical analysis was also performed using custom routines written in Python. To test whether the response characteristics demonstrate age-dependent changes for each of defined characteristics, the Spearman correlation coefficient with associated p-value was calculated. In addition, we used one-sided Wilcoxon rank sum test for comparisons in two groups of P8–12 (n = 13) and P13–16 (n = 10) animals, separated by the onset of low-threshold hearing (P13) as the crucial milestone of hearing development^10^, and representing the high- and low-threshold periods, respectively. The results of this test (Supplementary Table 1) were largely in agreement with the age-dependent differences obtained using Spearman correlation test. To compare relative spatial localization of different types of responses (and also P and N response components) we first calculated the average distances between peaks and then applied the Rayleigh test of uniformity to reveal whether there is a specific direction in which one peak is located relative to another. Second, we estimated the portion that the intersection of the half-width areas of two responses constitute from each of the half-width areas. The significance level was set at *p* < 0.05. Group data are expressed as mean ± SEM (calculated for all animals within a given group) unless otherwise indicated.

## Supplementary Information

Supplementary Information 1.

Supplementary Video 1.

## Data Availability

The data and code are available from the corresponding author upon request without any specific restrictions.
