# Supplementary material for: Bone conducted responses in the neonatal rat auditory cortex
Source: Sci Rep. 2021 Aug 18;11:16777. doi: 10.1038/s41598-021-96188-9 (PMC8373948; doi:10.1038/s41598-021-96188-9)
Supplement: Supplementary file 1 — Supplementary Information 1. [file 41598_2021_96188_MOESM1_ESM.pdf]

Bone-conducted responses in the neonatal rat auditory cortex

Roman Makarov[1], Mikhail Sintsov[1], Guzel Valeeva[1], Pavel Starikov[2], Dmitriy Negrov[2], Roustem Khazipov[1,3,*]

[1]Laboratory of Neurobiology, Kazan Federal University, Kazan, 420008, Russia
[2]The School of Electronics Photonics and Molecular Physics, Moscow Institute of Physics and Technology, Moscow, 117303, Russia
[3]INMED, INSERM UMR1249, Aix-Marseille University, Marseille, 13273, France
[*]Corresponding author: roustem.khazipov@inserm.fr

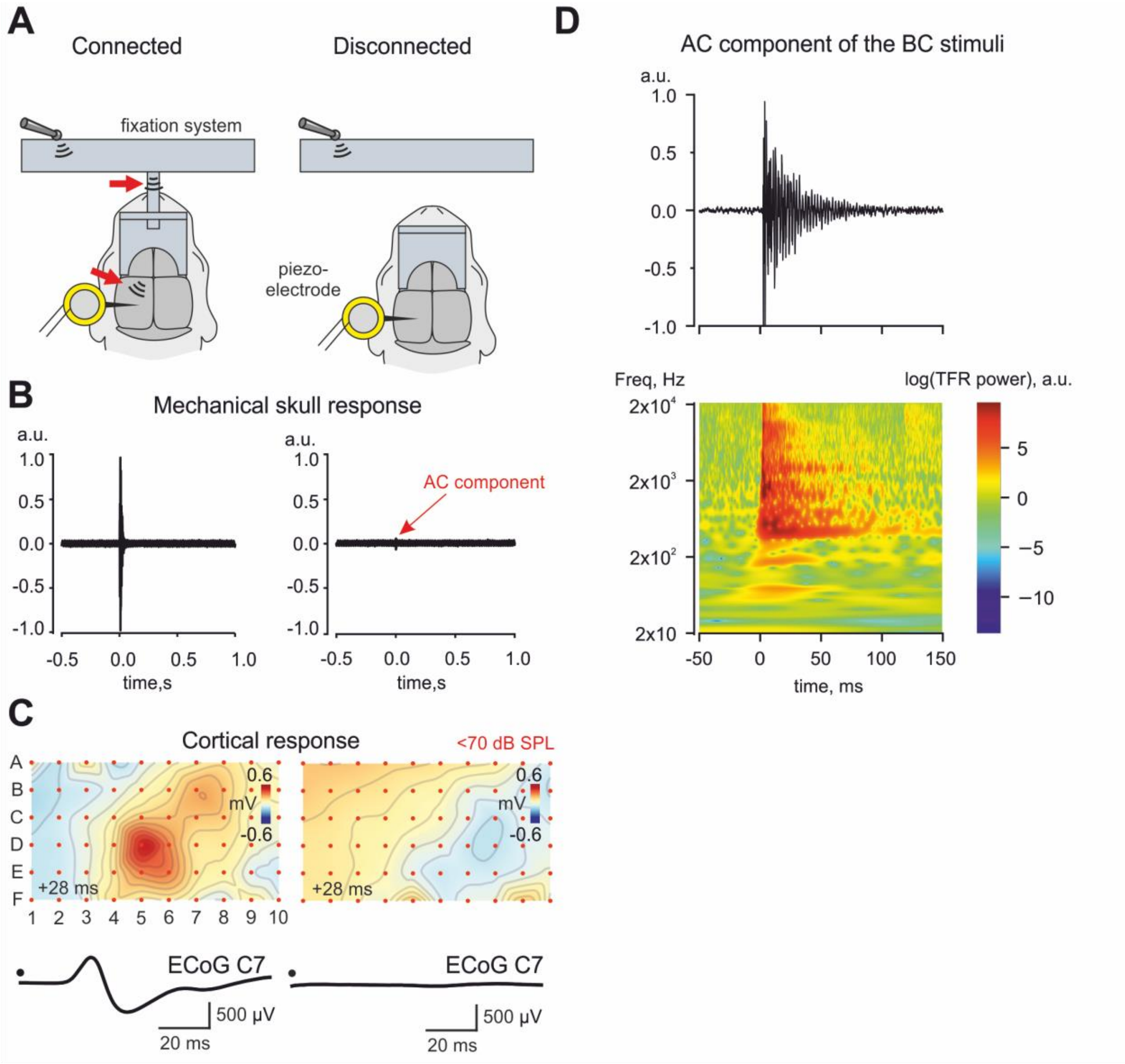

Figure S1. Measurements of stimulus intensity. (**A**) Experimental setup for measuring bone-conducted (BC) sound. A piezo-electrode with an attached needle was placed in contact with the surface of the skull. Mechanical stimuli were conducted through to a metal bar either connected (left) or not connected (left) with the skull. (**B**) Characteristic example of a mechanical skull response recorded by the piezo-electrode when the skull was connected (left) and not connected (right) to the bar. The amplitude is normalized to the maximal amplitude on the left plot. Note that the right plot reflects the AC component (red arrow) of the stimulus, which has a much smaller amplitude compared to the BC component on the left plot. The time scale is set relative to the stimulus onset (0.0 s). (**C**) Averaged cortical responses to mechanical stimuli when the skull was connected (left) and not connected (right) to the bar. Note that there is no detectable response when the skull is not connected to the bar. This figure illustrates that the applied stimuli indeed are conducted through the bones of the skull and have the AC component insufficient to evoke cortical responses. (**D**) A characteristic example of the AC component of BC stimuli recorded by a computer microphone (above) and its power spectrum in a range between 20 and 20000 Hz (below).

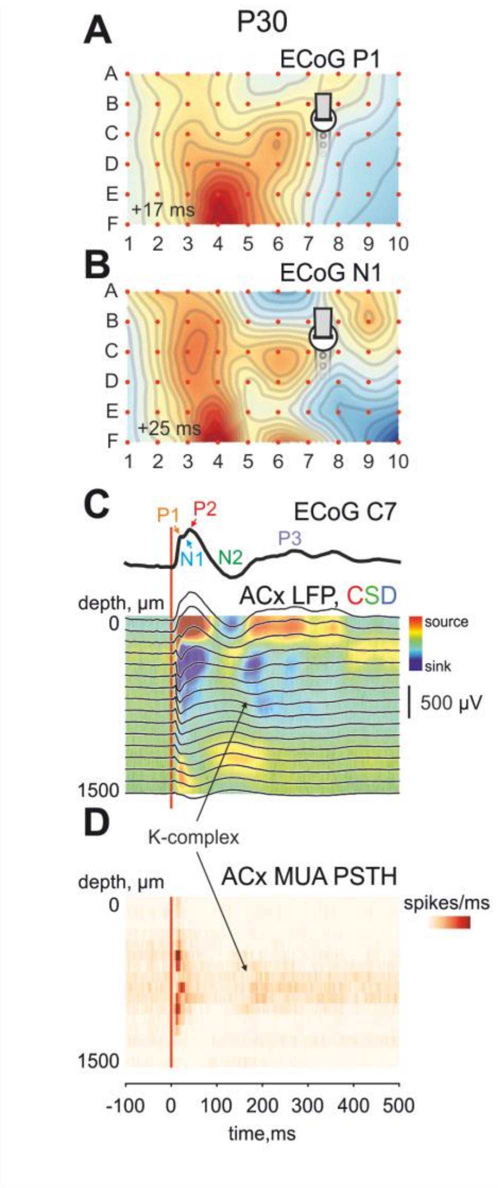

Figure S2. Coupled recordings with a µECoG electrode and an intracortical silicon probe in a P30 animal. (**A-B**) Spatial map of P (**A**) and N (**B**) component amplitudes. (**C**) Local field potential (LFP, black traces) overlaid on current source density (CSD) map at different depths of the auditory cortex. Above, the LFP signal from one of the closest channels of the ECoG electrode. (**D**) Multiunit activity (MUA) at different depths of the auditory cortex.

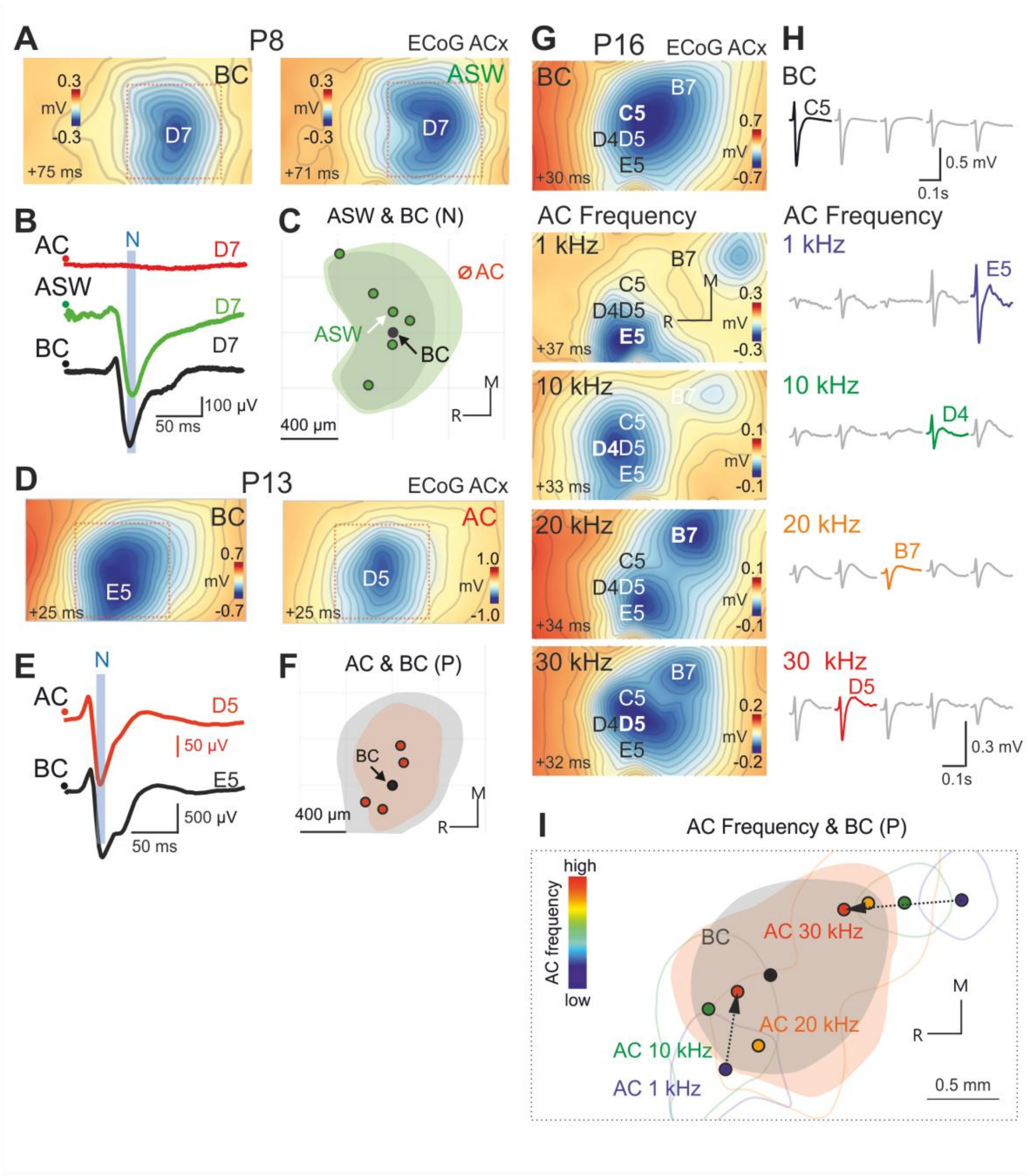

Figure S3. BC-evoked responses co-localize with the AC and ASW-evoked responses (The N component). (**A**) Spatial maps of the P component of BC and ASW responses in a P8 animal. The onset of the 2 ms time window in which the signal was averaged is shown in the lower-left corner. (**B**) Corresponding ECoG traces on the C6 electrode. Note that the AC stimulus does not evoke any detectable cortical response. (**C**) Positions of ASW responses (green dots) relatively to BC response (black dot) for N peaks. Half-width areas are shown for an animal on A and B (the correspondent peak is indicated by the white arrow). Pooled data from 6 P8-12 animals. (**D-F**) Responses evoked by BC and AC stimuli in a P13 animal. The layout is similar to (**A-C**).Pooled data from 4 P13-16 animals. (**G**) Spatial maps of BC (top) and AC-evoked (below) responses at different sound frequencies (1-30 kHz). The onset of the 2 ms time window in which the signal was averaged is shown in the lower-left corner. (**H**) ECoG traces on the channels with maximal amplitude chosen for each map in (**G**). (**I**) Positions of AC responses on different sound frequencies (indicated by color dots) relative to BC responses (black dot) for N peaks are shown for an animal on (**G** and **H**). Note that the half-width area of BC response (black fill) overlaps with the half-width area of AC response on 30 kHz (red fill), whereas two tonotopic gradients (black arrows) of AC responses converge towards the position of BC response.

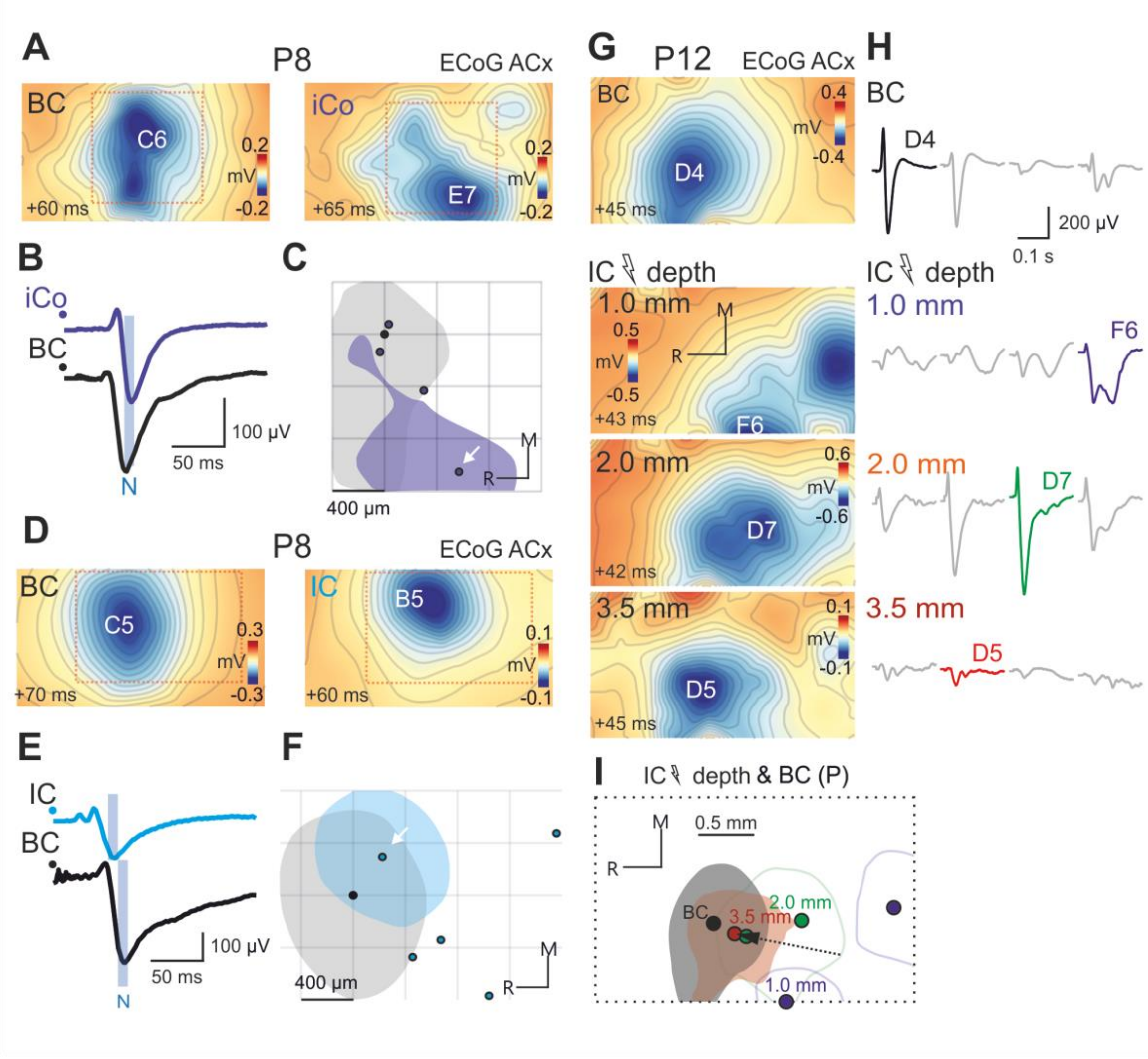

Figure S4. BC-evoked responses co-localize with iCO and IC-evoked responses (The N component). (**A**) Spatial maps of P component of BC and iCo- evoked responses in a P8 animal. The onset of the 2 ms time window in which the signal was averaged is shown in the lower-left corner. (**B**) ECoG traces on the channels with maximal amplitude chosen for each map in (**B**). (**C**) Positions of iCo-evoked responses (blue dots) relative to BC responses (black dot) for N peaks. Half-width areas are shown for an animal on A and B (the correspondent peak is indicated by the white arrow). Pooled data from 4 P8-16 animals. (**D-F**) Responses evoked by BC and IC stimuli in a P8 animal. The layout is similar to (**A-C**). Pooled data from 5 P8-15 animals. (**G**) Spatial maps of BC (top) and IC-evoked (below) responses at different depths of stimulation (1-3.5 mm). The onset of the 2 ms time window in which the signal was averaged is shown in the lower-left corner. (**H**) ECoG traces on the channels with maximal amplitude chosen for each map in (**G**). (**I**) Positions of IC-evoked responses on different depths of stimulation (indicated by color dots) relative to BC responses (black dot) for N peaks are shown for an animal on (**G**) and (**H**). Note that the half-width area of BC response (black fill) overlaps with the half-width area of IC response on 3.5 mm (red fill), whereas the tonotopic gradient (black arrow) of IC responses is directed towards the position of BC response.

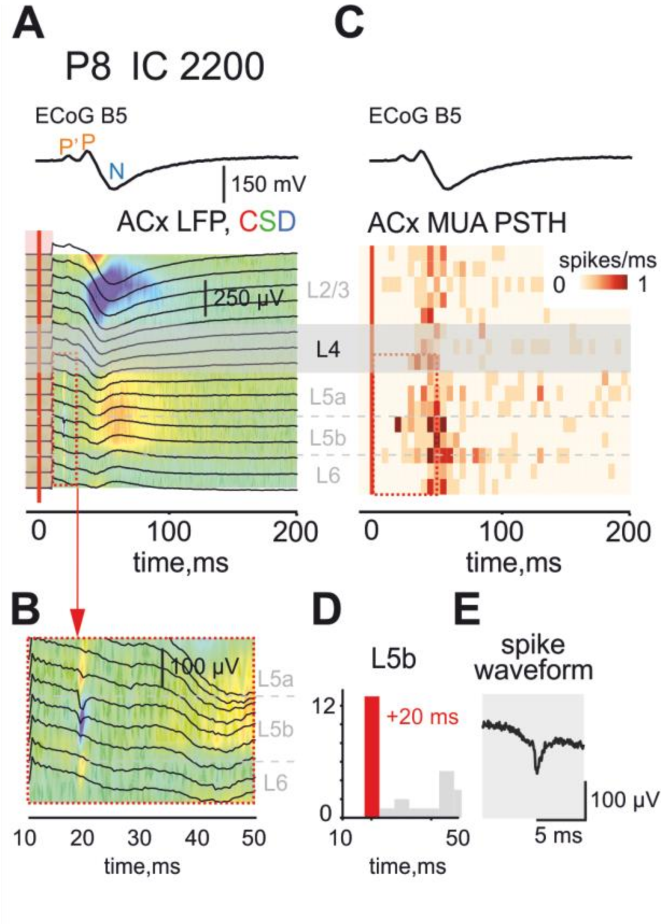

Figure S5. Antidromic activation of cortical L5 neurons by the stimulation of the IC. (**A**) Local field potential (LFP, black traces) overlaid on current source density (CSD) map at different depths of the auditory cortex. Above, the LFP signal from one of the closest channels of the µECoG electrode. Note the P' peak generated by the antidromic activation of L5 neurons. (**B**) A zoomed-in fragment from (**A**) with the channels containing the antidromic response. (**C**) Multiunit activity (MUA) at different depths of the auditory cortex. (**D**) Histogram of MUA on the initial 50 ms after the stimulus onset. Note the peak of the antidromic spikes on 20 ms. (**E**) An example of a characteristic spike waveform on a channel in L5.

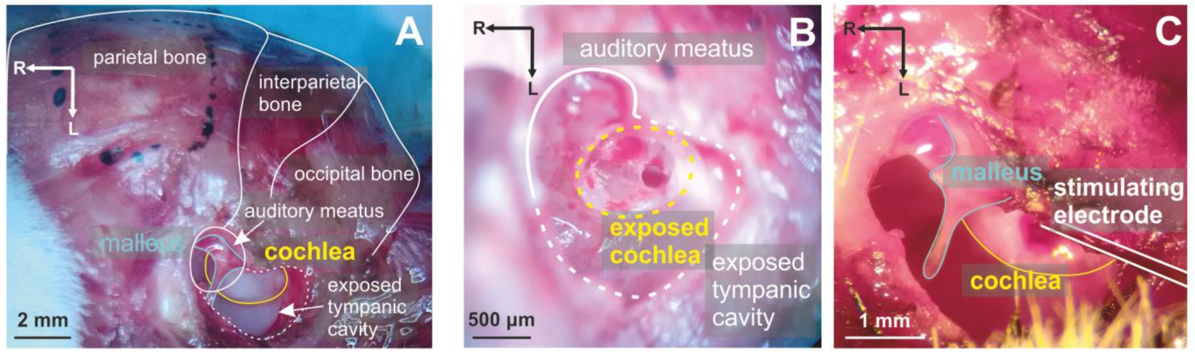

Figure.S6. Cochlear stimulation and ablation. (**A**) Surgical approach to the ear structures.R, rostral, L, lateral. (**B**) Exposure of the cochlear interior by perforating the cochlear wall. (**C**) Cochlear stimulation using a bipolar electrode.

Supplementary Table 1. Results of the Wilcoxon rank sum test									
parameter	P8-12 (n=13)				P13-16 (n=10)				p-value
	P_{25}	M1	P_{75}	R1	P_{25}	M2	P_{75}	R2	
P peak latency, ms	32.0	33.0	40.0	199.0	23.5	26.0	27.75	77.0	**0.003**
N peak latency, ms	48.0	51.0	60.0	200.0	35.5	39.5	41.5	76.0	**0.002**
P-N delay, ms	15.0	18.0	20.0	196.5	11.25	12.5	15.0	79.5	**0.006**
P peak amplitude, mV	75.0	120.0	200.0	127.5	155.0	275.0	337.5	148.5	**0.040**
N peak amplitude, mV	193.0	300.0	387.0	130.5	330	500.0	752.5	145.5	0.059
P-N distance, μm	201.0	266.0	470.0	147.0	232.25	342.5	373.75	129.0	0.302
P-N angle, rad	-1.09	-0.81	-0.29	138.0	-0.69	-0.55	-0.28	138.0	0.142
P half-width area, mm^2	0.44	0.5	0.66	117.0	0.65	0.89	1.22	159.0	**0.007**
N half-width area, mm^2	0.84	1.04	1.19	141.5	0.97	1.19	1.57	134.5	0.192
(P&N overlap) / P half-width area	0.75	0.91	0.94	197.5	0.49	0.66	0.72	78.5	**0.004**
(P&N overlap) / N half-width area	0.42	0.44	0.61	154.0	0.38	0.48	0.60	122.0	0.458

Supplementary table 1. Results of the one-sided Wilcoxon rank sum test for independent groups of animals representing high-threshold P8-12 (n=13) and low-threshold P13-16 (n=10) peridos. P_{25} , P_{75} - 25th and 75th percentiles, respectively. M - median, R - rank sum. Exact p-values were calculated. Significant p-values (< 0.05) are indicated in bold.
